# Supplementary material for: Characterization of habitat requirements of European fishing spiders
Source: PeerJ. 2022 Feb 1;10:e12806. doi: 10.7717/peerj.12806 (PMC8815374; doi:10.7717/peerj.12806)
Supplement: Supplemental Information 1 [file peerj-10-12806-s001.docx]

| **Site ID** | **Sampling date** | **Species** | **Nursery**  **count** | **Elevation**  **(m)** | **Latitude** | **Longitude** | **Surrounding** | **Surrounding forest** | **Vegetation type** | **Cattle**  **grazing** | **Water type** | **Water speed** | **Water vegetation** |
| --- | --- | --- | --- | --- | --- | --- | --- | --- | --- | --- | --- | --- | --- |
| 2 | 01/07/18 | none | 0 | 111 | 56.57 | 13.74 | infrastructure | deciduous | open_wet | 0 | river | slow | yes |
| 3 | 01/07/18 | none | NA | 149 | 56.54 | 13.81 | forest | coniferous | open_dry | 0 | creek | standing | no |
| 4 | 01/07/18 | none | NA | 154 | 56.53 | 13.77 | forest | coniferous | open_wet | NA | bog | standing | no |
| 5 | 02/07/18 | *D.*  *fimbriatus* | 0 | 64 | 56.29 | 12.94 | forest | deciduous | open_wet | 0 | bog | standing | yes |
| 6 | 03/07/18 | *D.*  *fimbriatus* | 0 | 43 | 56.16 | 13.70 | forest | deciduous | open_dry | 0 | creek | fast | yes |
| 7 | 04/07/18 | both | 8 | 43 | 56.16 | 13.70 | forest | deciduous | open_wet | 0 | river | slow | yes |
| 8 | 04/07/18 | none | 0 | 108 | 56.29 | 14.02 | forest | deciduous | Forest_  deciduous | 0 | river | slow | no |
| 9 | 04/07/18 | *D.*  *plantarius* | 1 | 1 | 56.03 | 14.15 | infrastructure | deciduous | open_wet | 0 | river | slow | yes |
| 10 | 04/07/18 | *D.*  *plantarius* | 3 | 2 | 56.00 | 14.26 | other | deciduous | open_wet | 1 | lake | standing | yes |
| 11 | 05/07/18 | *D.*  *plantarius* | 3 | 118 | 56.02 | 13.11 | infrastructure | deciduous | Forest_  deciduous | 0 | lake | slow | yes |
| 12 | 06/07/18 | both | 1 | 65 | 56.30 | 13.07 | forest | deciduous | Forest_  deciduous | 0 | creek | standing | yes |
| 13 | 06/07/18 | *D.*  *fimbriatus* | 4 | 193 | 56.39 | 12.96 | forest | deciduous | open_wet | 1 | bog | no | no |
| 14 | 08/07/18 | *D.*  *fimbriatus* | 0 | 49 | 59.52 | 13.49 | forest | deciduous | open_dry | 0 | creek | slow | no |
| 15 | 10/07/18 | *both* | 2 | 45 | 59.33 | 13.95 | fields | deciduous | open_wet | 1 | other | no | yes |
| 16 | 10/07/18 | *D.*  *plantarius* | 5 | 45 | 59.33 | 13.95 | fields | deciduous | open_wet | 0 | river | slow | yes |
| 17 | 10/07/18 | none | 1 | 135 | 59.48 | 14.11 | forest | coniferous | open_wet | 0 | lake | standing | yes |
| 18 | 11/07/18 | both | 1 | 98 | 59.57 | 13.05 | infrastructure | mixed | open_wet | 1 | lake | standing | yes |
| 19 | 11/07/18 | *D.*  *plantarius* | 8 | 45 | 59.35 | 13.46 | fields | deciduous | open_wet | 1 | other | standing | yes |
| 20 | 13/07/18 | *D.*  *fimbriatus* | 4 | 61 | 59.73 | 16.20 | infrastructure | deciduous | open_wet | 0 | other | no | no |
| 21 | 13/07/18 | none | 0 | 71 | 59.79 | 16.17 | forest | deciduous | open_wet | 0 | lake | standing | yes |
| 22 | 14/07/18 | *D.*  *fimbriatus* | NA | 34 | 60.49 | 17.36 | forest | mixed | open_wet | 0 | bog | no | yes |
| 23 | 17/07/18 | *D.*  *plantarius* | 0 | 25 | 59.36 | 15.96 | forest | deciduous | Forest_  deciduous | 0 | river | slow | yes |
| 25 | 27/07/18 | *D.*  *fimbriatus* | 1 | 289 | 61.39 | 11.18 | forest | coniferous | Forest_  pine | 0 | lake | standing | yes |
| 26 | 25/07/18 | *D.*  *fimbriatus* | 2 | 294 | 61.39 | 11.18 | forest | coniferous | open_wet | 0 | lake | standing | no |
| 27 | 25/07/18 | *none* | 1 | 295 | 61.39 | 11.17 | forest | coniferous | Forest_  pine | 0 | lake | standing | no |
| 28 | 26/07/18 | *D.*  *fimbriatus* | 0 | 251 | 61.38 | 11.19 | forest | coniferous | open_wet | 0 | lake | standing | yes |
| 30 | 09/08/18 | *D.*  *fimbriatus* | 0 | 216 | 57.10 | 14.57 | forest | coniferous | open_wet | 0 | bog | no | no |
| 31 | 10/08/18 | none | 0 | 185 | 57.12 | 14.57 | infrastructure | deciduous | open_wet | 0 | lake | standing | yes |
| 32 | 15/08/18 | none | 0 | 256 | 61.12 | 11.52 | fields | coniferous | open_wet | 0 | river | slow | yes |
| 33 | 15/08/18 | *D.*  *fimbriatus* | 1 | 234 | 61.16 | 11.47 | forest | coniferous | open_wet | 0 | lake | standing | yes |
| 24 | 02/08/18 | *D.*  *fimbriatus* | 1 | 218 | 60.88 | 11.57 | forest | coniferous | open_wet | 0 | lake | standing | yes |
