# Supplementary material for: Characterization of habitat requirements of European fishing spiders
Source: PeerJ. 2022 Feb 1;10:e12806. doi: 10.7717/peerj.12806 (PMC8815374; doi:10.7717/peerj.12806)
Supplement: Supplemental Information 3 [file peerj-10-12806-s003.docx]

| Model | negLogLike | delta | AIC | AICwt |
| --- | --- | --- | --- | --- |
| ~ sampling_type ~ 1 | 89.36 | 0.00 | 202.71 | 0.45 |
| ~1 ~ Cattle_grazing + type | 90.95 | 1.18 | 203.9 | 0.25 |
| ~ rCrossingStr + temperature + cloudyness + sampling_type ~ 1 | 92.86 | 3.00 | 205.71 | 0.1 |
| ~ temperature ~ 1 | 89.36 | 4.00 | 206.71 | 0.06 |
| ~ shade ~ 1 | 92.57 | 4.43 | 207.14 | 0.05 |
